# Analysis of High-Speed Cutting Surface Layer Formation and Oxide Layer Thickness Prediction of Titanium Alloy (Ti6Al4V)

**DOI:** 10.3390/ma18133160

**Published:** 2025-07-03

**Authors:** Chenyu Wang, Changyou Li, Huihui Miao, Zhi Tan, Wei Sun

**Affiliations:** 1 School of Mechanical Engineering and Automation, Northeastern University, Shenyang 110819, China; 2010087@stu.neu.edu.cn (C.W.); weisun@mail.neu.edu.cn (W.S.); 2 School of Mechanical Engineering, Taiyuan University of Science and Technology, Taiyuan 030024, China; miaohuihui@tyust.edu.cn; 3 General Technology Group Machine Tool Engineering Research Institute Co., Ltd., Beijing 100102, China

**Keywords:** surface-modified layer, Ti6Al4V, cutting speed, high-speed cutting

## Abstract

This study discusses the surface characteristics of titanium alloy Ti6Al4V during high-speed cutting, especially the effect of cutting speed on surface quality at different measuring scales. The experimental analysis shows that when the feed rate is 0.2 mm, and the detection scale is 1.2 mm, the surface roughness increases first and then decreases with the increase in the cutting speed. When the detection scale is 0.1 mm, the surface roughness continues to increase with the increase in the cutting speed. Based on the experimental results, this study adopted a research method combining experiment and simulation to intensely discuss the difference in the cutting speed’s mechanism of influence on surface quality under different detection scales. Based on the first principles, a prediction model for the oxide layer of high-speed cutting titanium alloy was constructed, and experiments verified the model’s accuracy. It was found that with the increase in the cutting speed, the cutting surface layer gradually formed a metamorphic layer, and the thickness of the oxide layer gradually increased, and it resultantly fell away. At the same time, the change in material microstructure and phase transition worked together to reduce the machining accuracy. In addition, the content of different components significantly affected the formation mechanism of the oxide layer, significantly increasing the Al content, which affected the oxygen diffusion activation energy and the oxide layer’s thickness.

## 1. Introduction

As a material widely used in aerospace, medical treatment, automotive, and other fields, titanium alloy (Ti6Al4V) has become a research hotspot due to its high strength, low density, corrosion resistance, and excellent biocompatibility [1,2,3]. However, Ti6Al4V is challenging to process, mainly characterized by its low thermal conductivity, high chemical activity, and small elastic modulus, resulting in low production efficiency, severe tool wear, poor machining surface quality, and other problems during processing [4]. With its advantages, such as high efficiency, high precision, and low cost, high-speed cutting technology has effectively solved these problems [5]. With the development of tool materials and structures, high-speed cutting technology has become an important part of advanced manufacturing technology. This technology can significantly improve the processing efficiency and surface quality and reduce the processing cost [6]. For titanium alloy materials, the cutting speed is higher than 70 m/min, which is a form of high-speed cutting [7].

The contact time between the tool and the workpiece is short when cutting at high speeds. The contact area is small, which makes it challenging to obtain the machining process information using experimental methods. In contrast, numerical simulation methods such as the finite element method are important in analyzing material behavior in high-speed cutting [8]. Li Xinjian et al. [9] adopted the response surface approximation method to optimize the Johnson–Cook parameters of the Ti6Al4V high-speed cutting simulation. They compared the chip morphology simulated by the optimized J-C parameters with the experimental results. Leonid Moiseevich Gurevich et al. [10] used Deform 3D software to simulate the high-speed cutting process of familiar and new sawing structures and discussed the influence of the tool angle on machining. Kundan Kumar Prasad et al. [11] conducted a study based on the finite element machining simulation to evaluate/calculate different machining responses during high-speed cutting.

In the metal cutting process, the interaction between the tool and the workpiece material generates a cutting force and cutting heat, especially under high-speed cutting conditions, resulting in a large amount of heat energy being released. Under the cutting force, the workpiece material undergoes plastic deformation, and the cutting heat causes an increase in material temperature [12,13,14]. Under the intense friction between the tool and the surface of the workpiece, the surface material of the workpiece is rapidly deformed, and the combined action of the cutting force and heat leads to the extrusion and slip of the internal grain of the material, which then elongates, distorts, overturns and even breaks [15]. At the micron level, a surface metamorphic layer with different physical and chemical properties from the titanium alloy matrix is formed below the machined surface. The morphology, size, and orientation of grains in the microstructure of this metamorphic layer have changed significantly [16]. The microstructure of the metamorphic layer is different from that of the base material zone. The cutting surface layer mainly comprises surface morphology, an oxidation layer, a thermal deformation layer, a work hardening layer, and a residual stress layer [17,18,19].

Due to the high hardness and brittleness of titanium alloy, its machining performance is poor, and it is difficult to obtain an ideal cutting surface, which has a crucial impact on improving the fatigue resistance of titanium alloy parts [20]. The fatigue failure of titanium alloy parts usually starts from the surface or surface area. Hence, research on the cutting surface characteristics is crucial to improving the fatigue resistance of titanium alloy parts [21]. The research of Dan Wang et al. [22] shows that the turning process of titanium alloy materials affects the surface structure of the material and, thus, affects its service life. The experimental results show that the fatigue cracks mainly originate from the cracks in the cutting layer. Matuszak Jakub et al. [23] discussed the influence of surface properties on fatigue life during milling, pointing out that the thickness of the hardened layer is negatively correlated with fatigue life. Zhao Xin et al. [24] analyzed the influence of surface integrity on fatigue life from surface morphology, microstructure, and residual stress through experiments involving the low temperature and dry cutting of Ti-5553 in liquid nitrogen. It was found that low-temperature cutting effectively improved the surface integrity of the sample, thus prolonging its fatigue life.

Regarding the formation and distribution characteristics of the cutting surface layer, Wu Shixiong et al. [25] designed low-speed and high-speed cutting experiments with different wear tools to study the microstructure, residual stress distribution, and hardness distribution of the surface and sub-surface layers of hardened steel after cooling at low temperatures. It was found that the microstructure changes caused by plastic deformation mainly occurred in the white layer and the transition layer. In contrast, the region below the transition layer did not change. Strong dislocation slip and plastic deformation are the key factors behind the formation of white and transition layers. Anhai Li et al. [26] used ABAQUS software to conduct orthogonal cutting experiments on TC4 titanium alloy and obtained the shear strain and strain rate history from the perspective of macroscopic deformation. They revealed the significant influence of crystal texture changes on the macroscopic properties of the machined surface. Li Junye et al. [27] adopted the standard neighbor analysis (CNA) method to identify the defect structure of the Ti-Al alloy workpiece. They studied the distribution and evolution process of the defect structure in the workpiece. It was found that during the nano-cutting process, some relatively stable defect structures were retained on the subsurface of the workpiece, forming a solid metal layer.

The review and analysis of the above-mentioned most advanced state indicates that the quality of the cutting surface layer has been widely studied. However, the formation characteristics of the high-speed cutting surface layer have not been thoroughly studied yet. Based on this, this study aims to systematically investigate the comprehensive impact of high-speed cutting on the surface layer of Ti6Al4V through a combination of multi-scale experimental characterization and first-principles numerical simulations and to clarify the correlation mechanism among surface quality, oxide layer formation, and phase transformation behavior at different cutting speeds. The main innovative points of this research are as follows: (1) A prediction model for the evolution of the oxide layer in the high-speed cutting of titanium alloys, considering the coupling of multiple physical fields, is established, providing a powerful theoretical tool for accurately predicting and controlling the oxidation state of the machined surface. (2) The differentiated influence laws of cutting speed on surface roughness at different measurement scales and its underlying mechanisms are revealed (such as thermal–force coupling, oxide layer shedding, phase transformation, and plastic deformation). (3) The significant regulatory effect of the content of key elements on the kinetics of oxide layer formation is clarified. (4) The influence of the mechanism of phase transformation (α→β) and microstructure changes induced by high-speed cutting on the performance of the surface layer was analyzed. The purpose of this study is to deeply analyze the influence of high-speed cutting on the surface layer morphology of Ti-6Al-4V cutting. Through a combination of experiments and numerical simulations, the influence of cutting speed on surface roughness, surface morphology, surface layer morphology, and modified layer is explored. The research results will provide theoretical guidance and optimize processing parameters for the high-speed cutting of Ti-6Al-4V, thereby improving processing efficiency and surface quality.

## 2. Experimental Material and Procedure

### 2.1. Cutting Surface Quality Test

**Figure 1:**
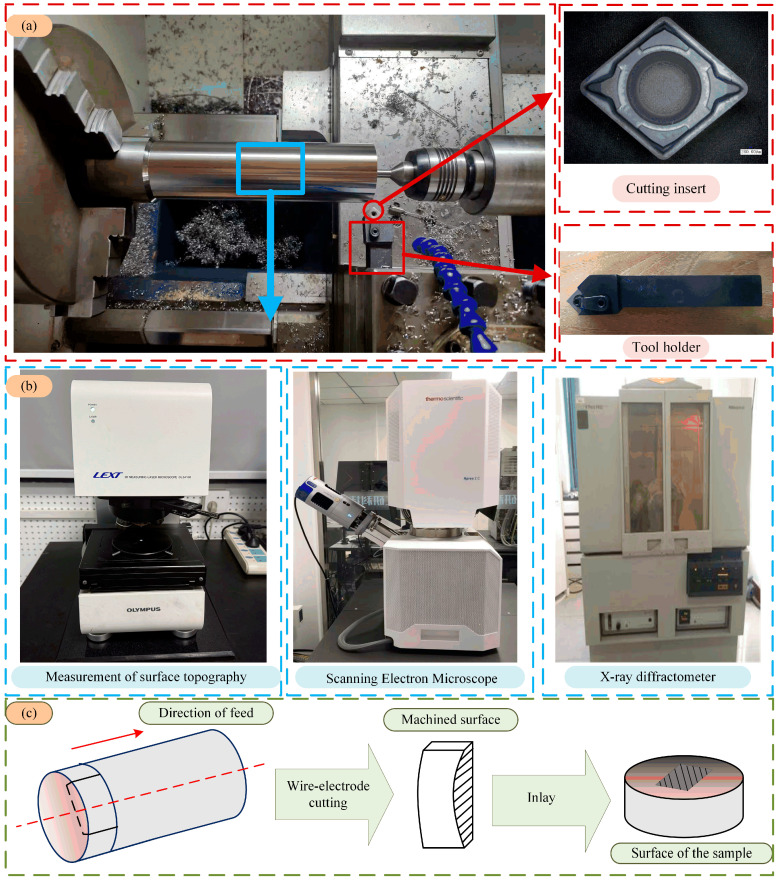
Schematic diagram of surface sample preparation for surface quality inspection. (**a**) Cutting processing test. (**b**) Cutting surface inspection equipment. (**c**) The process of preparing the surface sample for cutting.

The dry-cutting experiment was conducted on the CNC lathe C5075, produced by Eastern CNC (Daisen) Co., Ltd. (Dalin, China), as shown in Figure 1a. The cutting scheme is presented in Table 1. The tools used in this test were complex alloy tools from Sandvik. The tool handle number was PSSNR2020AK12, and the cutting insert number was SNMG 120408-23. The cutting scheme is shown in Table 1. The test material was a titanium alloy Ti6Al4V annealed bar with a total length of 300 mm and a diameter of 70 mm. The titanium alloy Ti6Al4V is α + β phase. The chemical composition and performance parameters of Ti6Al4V are shown in Table 2 and Table 3.

A laser confocal scanning microscope (LSCM, LEXT OLS4100) was used to observe the surface morphology, roughness, and microstructure. To deeply explore the microstructure of the metamorphic layer on the machined surface, the machined workpiece was further processed to prepare the metallographic sample, and the preparation process is shown in Figure 1c. The field emission scanning electron microscope (SEM) model Apreo 2C, produced by Thermo Fisher Technology Co., Ltd. (Shanghai, China), was used to observe the surface morphology after slicing. In addition, X-ray diffraction (XRD) analysis of the surface layer was carried out using an X-ray diffractometer (Cu-Kα radiation) produced by Philips of the Netherlands to evaluate the surface phase transformation.

### 2.2. Cutting Process Parameter Detection Experiment

**Figure 2:**
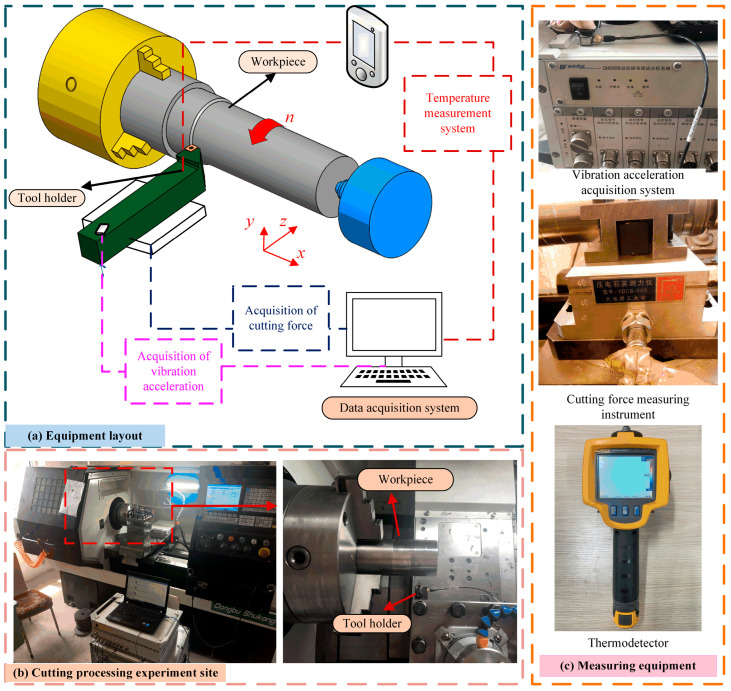
Diagram of cutting test setting.

During the dry-cutting experiment, the cutting process parameters were monitored. The layout of the monitoring equipment is shown in Figure 2a. During the cutting process, experiments were conducted using a three-phase cutting force measuring instrument, a cutting displacement sensor, and an infrared thermal imager. The experimental equipment is shown in Figure 2c, and the experimental plan is presented in Table 1.

## 3. Experimental Results and Analysis

### 3.1. Effect of Cutting Speed on Cutting Surface

**Figure 3:**
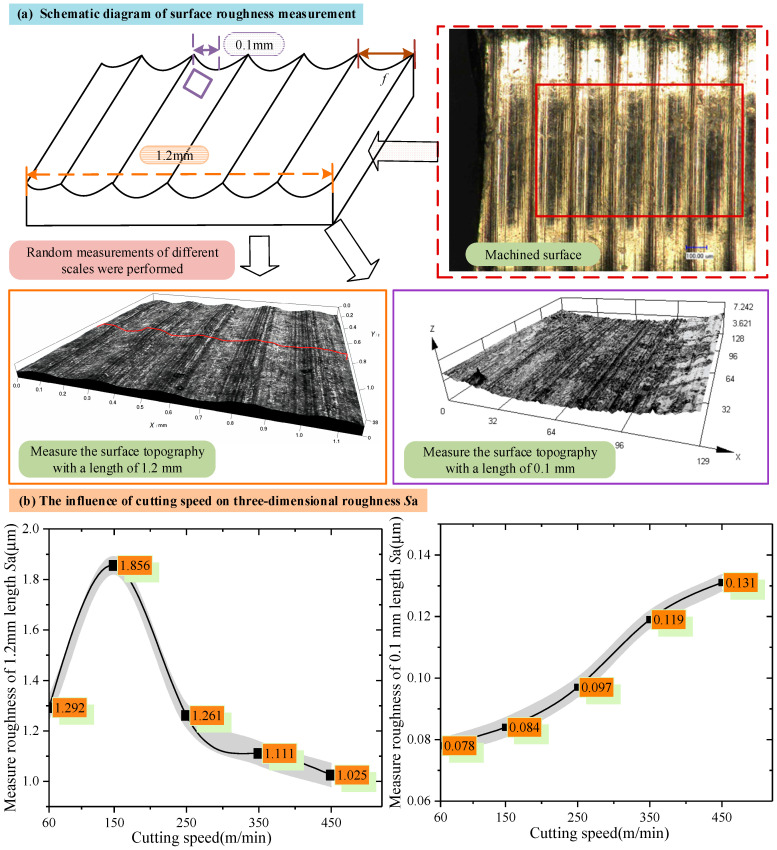
Experimental results of the effect of cutting speed on surface roughness.

Three positions were randomly selected for surface quality inspection, with the inspection scales being 1.2 mm and 0.1 mm, respectively. The results are shown in Figure 3a. The test results of three-dimensional surface roughness and surface topography under different cutting speeds are shown in Figure 3b. It can be observed from Figure 3b that when the detection scale is greater than the feed rate (1.2 mm), the three-dimensional roughness of the surface shows a trend of first increasing and then decreasing with the increase in the cutting speed. At a larger detection scale, the influence of cutting speed on surface quality is rather complex. There may be a combined effect of multiple factors, resulting in the variation in three-dimensional surface roughness presenting nonlinear characteristics. When the detection scale is less than the feed rate (0.1 mm), the three-dimensional surface roughness increases with the increase in the cutting speed. It is indicated that at a smaller detection scale, the increase in the cutting speed will directly affect the microscopic accuracy of the surface, resulting in a continuous increase in the three-dimensional roughness of the surface.

**Figure 4:**
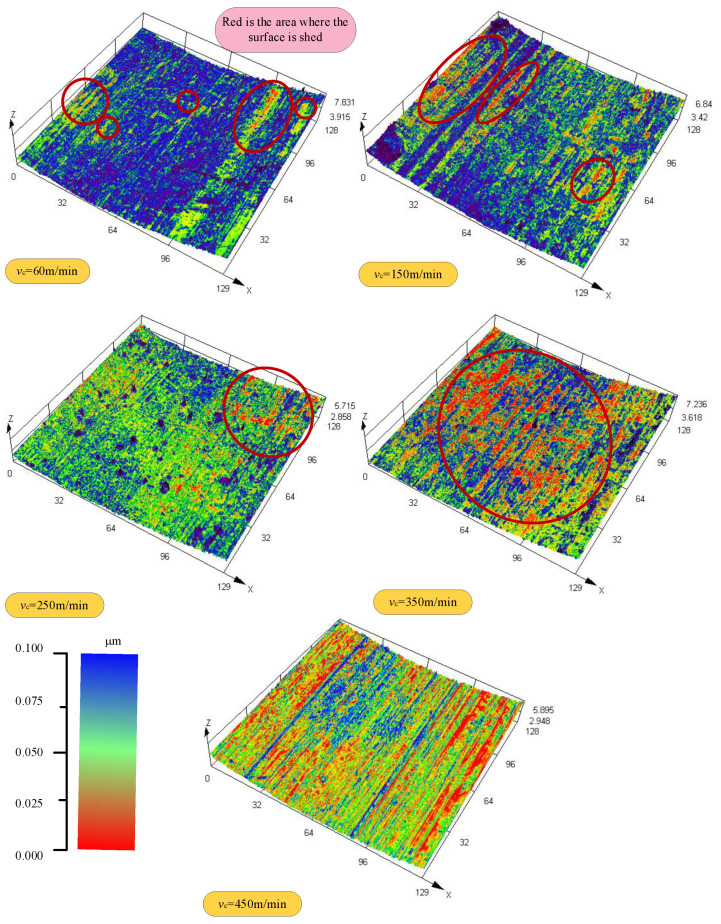
The cutting surface topography changes with the cutting speed.

Under different cutting speeds, the cutting surface was randomly selected to detect the surface topography with a scale of 0.1 mm. The detection results are color-coded and rendered according to the surface’s roughness, as shown in Figure 4. Red represents low roughness, and blue represents high roughness. By observing graphic data, the influence of different cutting speeds on the topography of the cutting surface is evident. When the cutting speed is 60 m/min, the surface peeling area is relatively small, and the red area is dispersed, reflecting a slightly better surface quality and no apparent shedding phenomenon. With the cutting speed increasing to 150 m/min, the surface shedding area increases, the red area becomes concentrated, the surface quality decreases and the shedding phenomenon is clear. When the cutting speed is further increased to 250 m/min, the surface shedding area is further increased, the red area is concentrated, the surface quality further deteriorates, and the shedding phenomenon is severe. When the cutting speed is 350 m/min, the surface shedding area increases significantly, the red area is widely distributed, the surface quality decreases significantly, and the shedding phenomenon is severe. Finally, when the cutting speed reaches 450 m/min, the surface shedding area reaches its maximum size, the red area almost covers the entire surface, the surface quality is inferior, and the shedding phenomenon is severe.

### 3.2. The Effect of Cutting Speed on the Cutting Surface Layer

**Figure 5:**
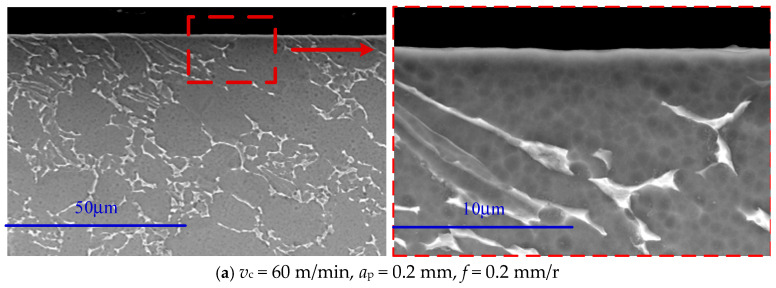

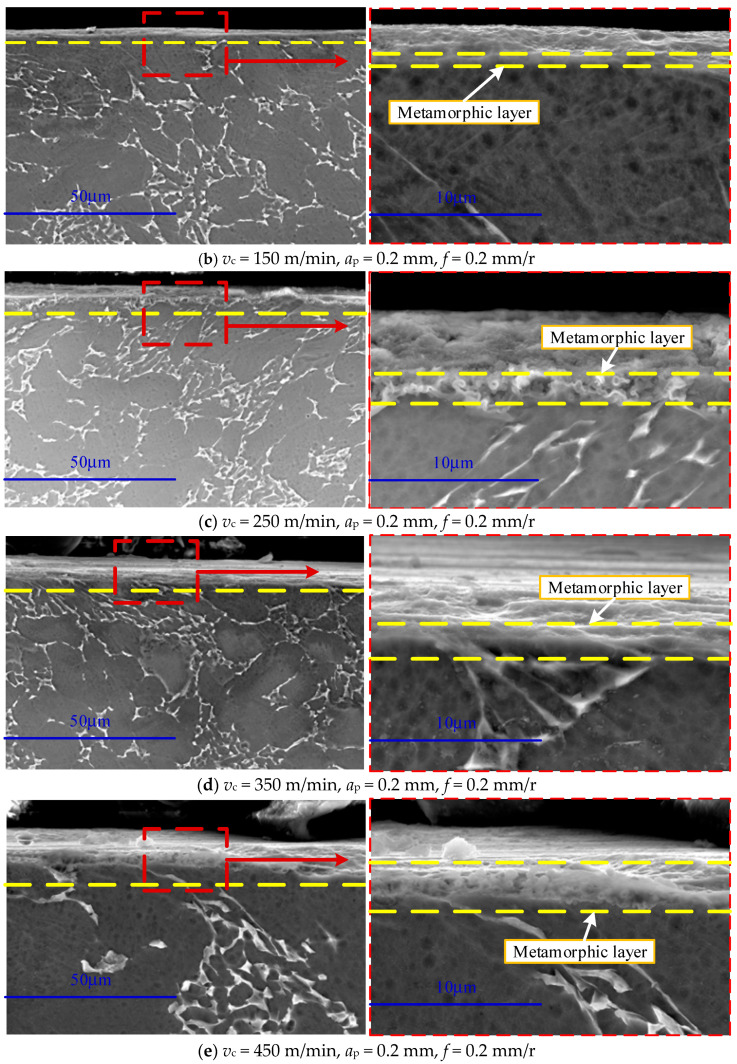
Cutting surface morphology.

To thoroughly investigate the impact of high-speed cutting on the cutting surface layer, this study focuses on the surface-shedding phenomenon that occurs when the cutting speed is increased, aiming to analyze its causes. This study utilizes scanning electron microscopy (SEM) to examine the surface after cutting. Figure 5 shows the morphological characteristics of the cutting surface layer of the Ti6Al4V alloy at different cutting speeds (60 m/min, 150 m/min, 250 m/min, 350 m/min, 450 m/min). When the cutting speed is 60 m/min (Figure 5a), no newly formed material is observed on the surface. Apart from a small amount of grain-tearing, the microstructure of the surface is not significantly different from that of the substrate, indicating that the cutting speed has little effect on the surface structure at this time. When the cutting speed is increased to 150 m/min, new substances with different properties from the substrate appear on the surface, forming a metamorphic layer of a specific thickness. With the further increase in the cutting speed, the thickness of the metamorphic layer exhibits a gradually increasing trend. This phenomenon suggests a significant positive correlation between the increase in cutting speed and the thickness of the metamorphic layer.

**Figure 6:**
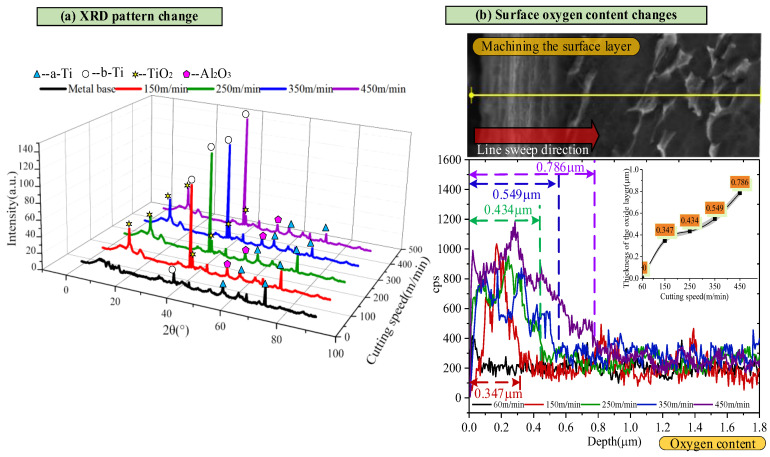
Surface oxygen content changes with cutting speed.

In order to identify the specific composition of the cutting metamorphic layer, X-ray diffraction (XRD) technology was used to analyze the composition of the metamorphic layer. The correspondence between the diffraction angles and the diffraction intensities of *α* and *β* phases determines the phase types corresponding to each diffraction peak. The analysis results (Figure 6a) show that the uppermost layer of the metamorphic layer is mainly composed of rutile-type TiO_2_ and a small amount of Al_2_O_3_. It is worth noting that, compared with the base material, the diffraction peak of the machined surface shifts to the right, which is attributed to the lattice distortion of the titanium alloy machined surface. Further observation shows that the peak right-hand deviation distance is relatively small when the cutting speed is 150 m/min, 250 m/min, and 350 m/min. However, the peak shift to the right is more significant when the cutting speed increases up to 450 m/min. According to Bragg’s law, the greater the *θ* value, the more minor the crystal plane spacing is (D). This indicates that the cutting process causes lattice shrinkage and generates residual compressive stress. In summary, factors such as high-temperature softening, microstructure plastic deformation, and workpiece material phase transformation synergize the machined surface layer. In addition, according to the XRD pattern, there were peaks in both TiO_2_ and Al_2_O_3_, and the number of peaks in TiO_2_ was higher than in Al_2_O_3_.

Due to the limited depth of XRD detection, this study draws on previous research results and considers the continuous oxygen penetration of titanium alloys under high-temperature oxidation conditions [25]. Therefore, a scanning electron microscope (SEM) was used to conduct the line scanning of the surface samples to determine the thickness of the oxygen permeation layer accurately. The scanning results (Figure 6b) clearly show that as the cutting speed increases, the thickness of the oxygen-permeable layer gradually increases. Its variation trend is the same as that of the thickness variation trend of the deteriorated layer detected by the scanning electron microscope, as shown in Figure 5. This result strongly suggests that the infiltration of oxygen causes the deterioration of the layer during the high-speed cutting process.

**Figure 7:**
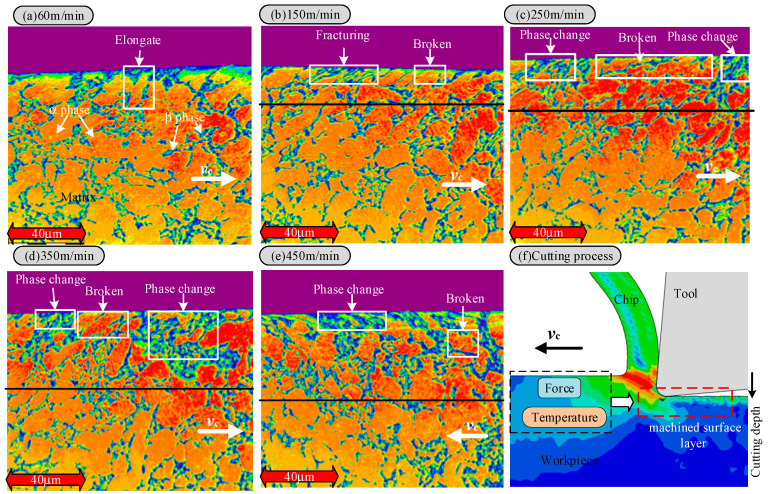
The surface metallographic structure changes with cutting speed.

In order to further analyze the characteristics of the cutting metamorphic layer, the metallographic analysis of the cutting surface was carried out in this study. Figure 7a shows the cutting surface metallographic structure at a 60 m/min cutting speed. It can be seen from the figure that the surface grain elongates significantly at this velocity, forming a plastic deformation zone with pronounced directivity and a high dislocation density. No prominent phase change area indicates that the work hardening layer is the primary layer at this time. This phenomenon is due to the large cutting force and rapid heat loss experienced under low-speed cutting conditions, and mechanical deformation plays a leading role [28]. The high stress causes grain boundary slip and dislocation accumulation to form the work-hardened layer, while the residual stress is concentrated at the boundary of the metamorphic layer. When the cutting speed is increased to 150 m/min, the surface layer has an obvious metamorphic boundary, the surface grain is partially refined, and the dynamic recrystallization of small grains can be seen. This situation indicates that the work hardening and local recrystallization zones coexist at this time. With the increase in the thermo-mechanical coupling, the temperature of some regions reaches the critical value of dynamic recrystallization. When the cutting speed is further increased to 250 m/min, the thickness of the metamorphic layer continues to increase, the work-hardened layer almost disappears, and the phase change layer begins to dominate, with no cracks or holes appearing. It shows that the material is austenitized under high-speed cutting and high temperatures, and then martensitic transformation occurs during the cooling process [29]. When the cutting speed reaches 350 m/min and 450 m/min, there are more phase transitions in the surface layer, the thickness of the metamorphic layer increases further, and the broken phase also increases.

As shown in Figure 6a, the XRD results indicate that Ti6Al4V is a typical two-phase (α + β) alloy. The α phase has a dense hexagonal structure, while the β phase has a body-centered cubic structure. Compared with the matrix, the content of the α phase on the machined surface decreases, and the content of the β phase increases accordingly. In the Ti6Al4V alloy, there are four α-Ti independent sliding systems and as many as 12 β-Ti independent sliding systems. Due to the existence of more independent slip systems in β-Ti, plastic deformation is more likely to occur during the cutting process. With the continuous increase in cutting speed and the rise in cutting temperature, phase transformation occurs on the machined surface. A large number of α phases transform into β phases, intensifying the degree of plastic deformation in the cutting layer [30]. Therefore, as the cutting speed increases, the workpiece surface undergoes thermal softening and phase transformation processes, resulting in grain distortion, elongation, and an increase in the depth of the plastic deformation layer. In addition, an increase in the β phase content usually indicates a decrease in the overall yield strength of the material (especially at high temperatures), which reduces the fatigue strength. Severe plastic deformation introduces relatively high residual tensile stress in the surface layer, which is typically a favorable location for the initiation of fatigue cracks. The phase transformation process itself may introduce microscopic defects or cause stress concentrations at the phase interface, becoming a potential source of fatigue cracks.

An overly deep plastic deformation layer may imply that a larger volume of the material is in a potentially damaged state [31]. As the Ti6Al4V alloy is a two-phase (α + β) titanium alloy, there are significant differences between the α phase and the β phase in terms of crystal structure and physical and mechanical properties. Therefore, it is essential to understand its phase transformation during processing. High-stress and rapid-heating conditions can effectively promote the transformation from the α to β phase. However, the phase transformation mechanism during the cutting process differs from that of conventional heat treatment, primarily due to the following aspects: First, the material is subjected to mechanical loads during the cutting process, accompanied by severe deformation. Second, the cutting process is characterized by rapid heating and cooling. Thirdly, there is a complex heat exchange between the processed surface and the environment. These factors work together to make the phase transformation mechanism in the cutting process more complex and unique [32].

**Figure 8:**
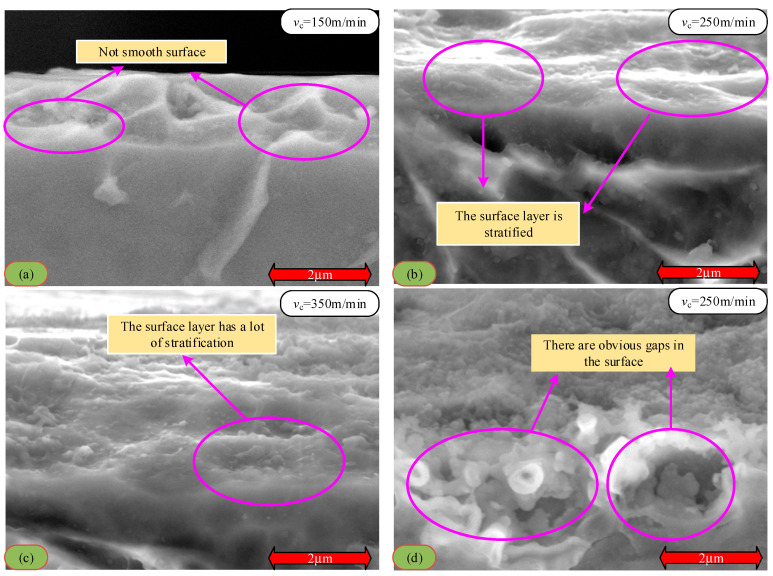
Partial magnification of titanium oxide layer. ((**a**) *v*_c_ = 150 m/min, (**b**) *v*_c_ = 250 min/min, (**c**) *v*_c_ = 350 m/min, (**d**) *v*_c_ = 250 m/min).

With the increase in cutting speed, the cutting area temperature increases significantly. This phenomenon is attributed to the extensive conversion of mechanical energy to thermal energy during the cutting process and the poor thermal conductivity of titanium alloy Ti6Al4V, which causes heat to accumulate in the cutting area. At high temperatures, titanium alloys react chemically with oxygen in the environment, resulting in surface oxidation. With a further increase in cutting speed, the cutting temperature rises, the oxidation reaction intensifies, and the oxide layer thickness increases correspondingly. The formation of the oxide layer changes the properties of the surface material and weakens its bonding strength with the base material. When the thickness of the oxide layer reaches a certain degree, under the action of cutting force, the oxide layer quickly falls off, resulting in defects on the machined surface and affecting the machining accuracy. This phenomenon is the main reason for the peeling phenomenon of the surface layer in Figure 4. The enlarged analysis of the metamorphic layer on the cutting surface shows that the surface topography presents an irregular structure (Figure 8a), stratification phenomenon (Figure 8b,c), and pores even appear in some areas (Figure 8d). All these factors reduce the binding force between the metamorphic layer and the substrate, thus causing the surface layer to fall off.

In addition, during the high-speed cutting process, the microstructure of titanium alloy Ti6Al4V undergoes a series of changes. The increased cutting speed enhances the surface material’s thermal and mechanical coupling effect, resulting in deformation, refinement, and even dynamic grain recrystallization. These changes in microstructure affect the material’s mechanical and processing properties. For example, the grain may have higher hardness and strength after dynamic recrystallization [33]. However, it is also more likely to crack and fracture during the cutting process, resulting in the loss of the surface layer and reduced processing accuracy. In addition, the phase transition also leads to a decline in surface accuracy; as the cutting speed and cutting temperature increase, the α phase gradually transforms into the β phase. The β phase has more slip systems and is prone to plastic deformation. During the phase transition process, the volume of the material changes, resulting in residual stress. These residual stresses under the combined action of the cutting force and oxide layer may cause the surface layer to fall off, affecting the quality and accuracy of the machined surface.

In summary, in the high-speed cutting process of titanium alloy Ti6Al4V, the increase in cutting speed leads to a rise in the cutting temperature, alongside the thickening and shedding of the oxide layer and changes in the material’s microstructure and phase transition.

**Figure 9:**
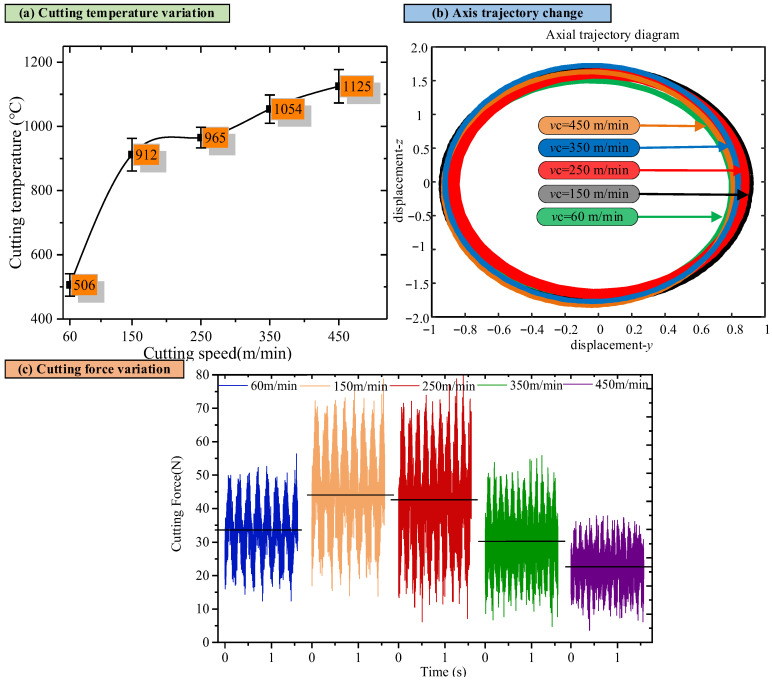
Cutting temperature, cutting force, and cutting vibration vary with cutting speed.

To further study the formation mechanism of the cutting metamorphic layer, the cutting force, cutting heat, and cutting vibration during the cutting process were systematically measured (Figure 9). The results show that with an increase in cutting speed, the cutting force and vibration acceleration initially increase and then decrease. This phenomenon can explain why the surface roughness fluctuates with the cutting speed at a larger detection scale (1.2 mm). The results show that, with an increase in cutting speed, the cutting force and vibration acceleration initially increase and then decrease. This phenomenon can explain why the surface roughness fluctuates with the cutting speed at a larger detection scale (1.2 mm). During low-speed cutting, as the cutting speed increases, the cutting force also increases. The increase in cutting force leads to an intensification of the contact deformation between the tool and the workpiece. During the cutting process, the cutting tool may generate slight displacements or vibrations, which can leave deeper scratches or ripples on the machined surface, thereby increasing the surface roughness [34,35].

Additionally, during low-speed cutting, chip formation is relatively slow. The friction between the chips and the rear face of the tool can also cause a certain degree of abrasion on the machined surface. As the cutting speed increases, this abrasion effect may intensify, thereby leading to an increase in surface roughness. When the cutting speed increases to a certain extent, the cutting force decreases, and the contact deformation between the tool and the workpiece reduces. Meanwhile, the rapid removal of chips during high-speed cutting reduces the scratching effect on the machined surface. The cutting process and the movement of the tool are more stable, which enables the formation of a smoother surface on the machined part, thereby reducing surface roughness. During high-speed cutting, the material softens at high temperatures, allowing it to cut into more minor chips easily. These chips have a reduced destructive effect on the machined surface and are also conducive to lowering the surface roughness. This nonlinear phenomenon is the result of the combined effect of various physical factors (such as the cutting heat, cutting force, tool–workpiece interaction, etc.) during the cutting process. They restrict and influence each other, causing the cutting force, vibration acceleration, and surface roughness at a larger detection scale (1.2 mm) to exhibit a trend of first increasing and then decreasing.

Furthermore, the increase in the cutting speed results in a rise in the cutting temperature, which, in turn, affects the formation of the surface oxide layer. Therefore, at a smaller detection scale (0.1 mm), a positive correlation exists between the change in surface roughness and the cutting temperature. When using an increased cutting speed in the growth and shedding of the metamorphic layer, it becomes uneven, resulting in a gradual increase in surface roughness.

### 3.3. Prediction of Oxide Layer Thickness

Based on the above analysis, the peeling of the Ti6Al4V cutting surface is mainly due to the high-temperature oxidation behavior of the material. In order to further study the formation mechanism of oxidation layer spalling, the first principles were used to predict the oxygen-permeable layer. In the case of the formation of oxide film on the metal surface, the sustainability of the oxidation reaction is limited by two main factors: one is the reaction rate of the oxidation reaction interface, which mainly involves the reaction rate of the metal matrix and oxide interface and oxide and gas interface. The second is the diffusion rate of the reactants participating in the oxidation reaction through the oxide film. It was found that the interfacial reaction rate is significantly higher than the diffusion rate of titanium ions and oxygen ions through the titanium oxide film. Therefore, the main control factor of the oxidation reaction rate of titanium alloy is the diffusion rate of titanium ions and oxygen ions through the oxide film. In the temperature range of 25 °C to 1200 °C, the oxidation rate of the titanium alloy is mainly controlled by the diffusion rate of oxygen ions through the oxide film. The diffusion rate of oxygen ions through the oxide film can be characterized by diffusion flux. Therefore, the first principles calculation method was used in this study to determine the diffusion flux.

According to Fick’s first law, the flux of a substance through a unit cross-sectional area perpendicular to the diffusion direction in unit time can quantitatively describe the process of a substance’s diffusion from a region of high concentration to a region of low concentration. The specific expression is shown in Equation (1):

(1) $$J=-D\frac{\partial C}{\partial X}=-D\left(\frac{\partial C}{\partial x}+\frac{\partial C}{\partial y}+\frac{\partial C}{\partial z}\right)$$

where *J* is the diffusive flux and $\frac{\partial C}{\partial x},\frac{\partial C}{\partial y},\frac{\partial C}{\partial z}$ is the concentration gradient of the diffuse layer in the *x*, *y*, *z* directions. *C* is the atomic concentration per unit volume and the negative sign indicates that diffusion is in the opposite direction to the concentration gradient. *D* is the diffusion coefficient.

In titanium alloy turning operations, the oxygen concentration in the oxidizing environment can be considered constant and does not vary with time; as the titanium alloy contains only trace amounts of oxygen internally, the oxygen concentration inside the titanium matrix can be considered zero. The oxygen concentration gradient, therefore, does not vary with the oxidation time and undergoes steady-state diffusion. The plane linearity of the oxide film is much greater than the thickness. Therefore, the oxygen concentration gradient exists only in the direction perpendicular to the oxide film, i.e., in the direction of the oxide film thickness, and has one-dimensional diffusion. Let the thickness of the oxide film be *x*, and the concentration of oxygen atoms be *C*_N_. Fick’s first law can be written as follows:

(2) $$J=-D\frac{0-C_{N}}{x}$$

This is according to the ideal gas equation of state:

(3) $$V_{2}=\frac{P_{1}V_{1}T_{2}}{P_{2}T_{1}}$$

*P*_1_, *V*_1_, and *T*_1_ are the standard pressure conditions, standard molar volume, and temperature conditions. *P*_2_, *V*_2_, and *T*_2_ are the pressure, molar volume, and temperature of the oxidizing environment during the turning process.

(4) $$n=\frac{10^{-3}}{V_{2}}$$

(5) $$C_{N}=2nN_{A}C=2\times 10^{-3}\frac{N_{A}C}{V_{2}}$$

where *N*_A_ = 6.02 × 10^23^/mol is the Avogadro constant.

The Arrhenius formula gives the diffusion coefficient as a function of temperature and diffusion activation energy:

(6) $$D=D_{0}\exp(-\frac{Q}{RT})$$

where *Q* is the diffusion activation energy; the particle must have the extra energy to overcome the energy barrier and achieve the particle jump from one equilibrium position to another. This part of the energy is called diffusion activation energy. *D*_0_ is the diffusion constant.

Under normal conditions, *P* = 0.1 MPa, *V* = 22.4 L/mol, and *T* = 273 K.

(7) $$J=0.24\frac{\alpha {a_{0}}^{2}v_{0}N_{A}CP}{xT}\exp\left(-\frac{Q}{RT}\right)$$

Let the oxidation rate of titanium alloy be *y*. Since the oxidation rate of titanium alloy is directly proportional to the diffusion flux of oxygen, the oxidation rate of titanium alloy can be expressed as follows:

(8) $$y\infty J=0.24\frac{\alpha {a_{0}}^{2}v_{0}N_{A}CP}{xT}\exp\left(-\frac{Q}{RT}\right)$$

where *y* is the oxidation rate of the turned titanium alloy, and *J* is the diffusion flux of oxygen through the oxide film. *C* is the oxygen concentration in the oxidizing environment. *T* is the cutting temperature. *x* is the thickness of the oxide film (cm). *P* is the atmospheric pressure. *Q* is the diffusion activation energy. *R* is the gas constant (8.314/mol·*K*). *α* is a factor depending on the crystal. α is a factor depending on the crystal’s geometry, while the body-centered and face-centered cubic crystal geometry α = 1. *a*_0_ is the crystal’s lattice constant, and *v*_0_ is the frequency of the particle vibrating in the equilibrium position of the lattice.

**Figure 10:**
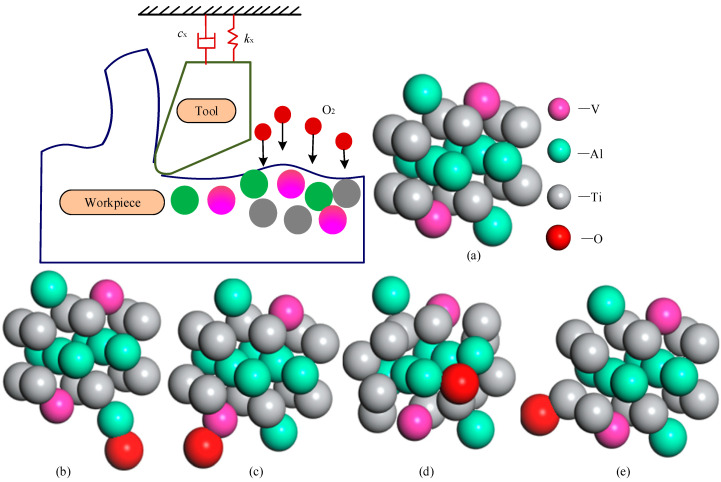
Model of oxygen migration in Ti6Al4V alloy system: (**a**) Ti6Al4V alloy system. (**b**) O migrates to Al. (**c**) O migrates to V. (**d**) O migrates to Ti1. (**e**) O migrates to Ti2.

Titanium ions and oxygen ions can move in the oxide film under the action of the electric field. At this time, it is difficult for electrons to move. Because electrons accompany ion movement, the growth of the oxide film depends on how electrons move in the film. In order to predict the thickness of the oxide layer, the diffusion activation energy of the oxygen atom in the Ti6Al4V system was calculated by the ab initio molecular dynamics method. Several forms of oxygen migration models in the Ti6Al4V alloy system selected in this paper are shown in Figure 10. All density functional theory calculations in this paper were carried out in the DMol3 package of Material Studio 2019 software. The ab initio molecular dynamics method is used to find the system’s optimal structure, and the initial structure’s influence can be ignored. The convergence standard of self-consistent field (SCF) calculations for total energy is 10^−5^ Hartree. The maximum force and displacement of the atom are 4 × 10^−3^ hartree/Å and 5 × 10^−3^ Å, respectively. The seaming value is 0.01 hartree to obtain reasonable convergence, and the charge and spin are set to 0.2 and 0.5, respectively. We used the all-electronic method for the geometric optimization and property calculation to optimize the structure in Perdew–Wang 91(PW91) Generalized Gradient Approximation (GGA) functional repetition. Double numerical polarization and diffusion functions (DNP) were used to treat the base group.

(9) $$\Delta G=\Delta H-T\Delta S=\Delta E+\Delta Z_{\mathrm{PVE}}+\int \Delta C_{P}dT-T\Delta S=\Delta E+\Delta H_{\mathrm{corr}}-T\Delta S$$

∆*G*, ∆*H*, ∆*E*, ∆*Z*_PVE_, ∆*C*_p_, ∆*S*, and ∆*H*_corr_ are the free energy, enthalpy, total energy, zero-point energies, heat capacity, entropy, and temperature-corrected enthalpy changes. The values of ∆*E*, ∆*S*, and ∆*H*_corr_ were obtained from the density functional theory (DFT) calculations.

**Figure 11:**
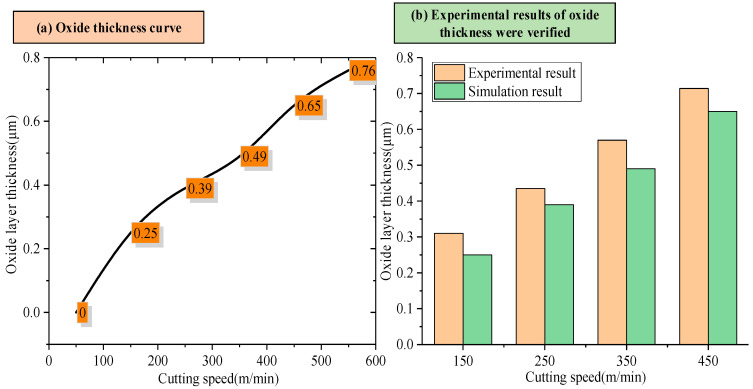
Effect of cutting speed on oxide layer thickness and experimental results.

The calculated diffusion of activation energy is substituted into Equation (8). When the oxygen flux is zero, assuming no further diffusion of oxygen atoms, x represents the thickness of the oxide layer. At different cutting speeds, the thickness of the oxide layer is shown in Figure 11a. In order to verify the accuracy of the oxide layer prediction model, the calculated oxide layer thickness was compared to that obtained by SEM line scanning, and the comparison results are shown in Figure 11b. Compared with the experimental results, the error of the oxide layer prediction model established in this paper is less than 15%, indicating that the model can predict the oxide layer thickness of high-speed cutting titanium alloy.

**Figure 12:**
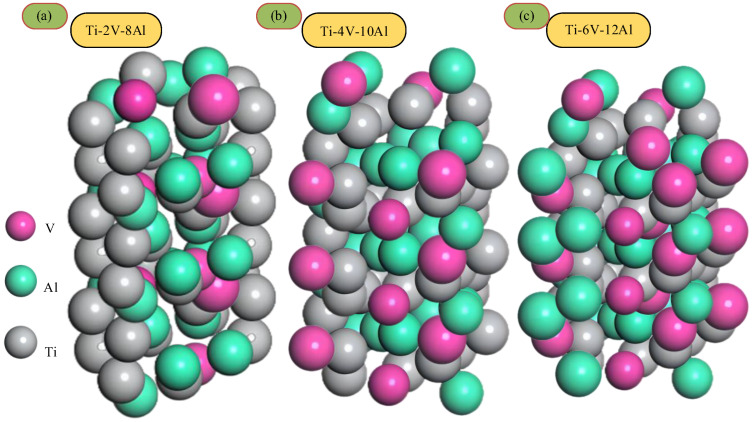
Several typical elements proportional to titanium aluminum alloy structure.

**Figure 13:**
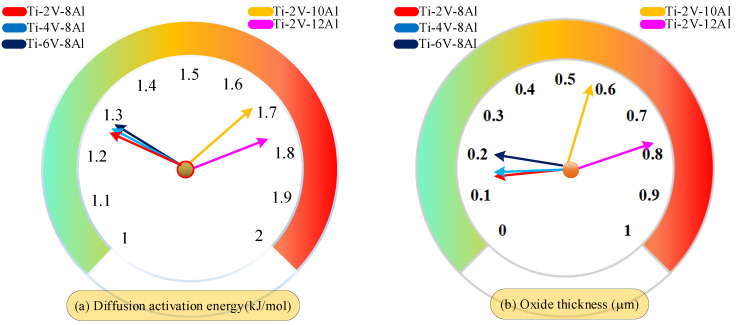
Comparison of effects of different element proportions on diffusion activation energy and oxide layer thickness.

In order to study the influence of different component contents in titanium alloy on the formation mechanism of the oxide layer further, simulation experiments, as shown in Figure 12, were designed to calculate the diffusion activation energy and oxide layer thickness of titanium alloy oxygen under different element proportions. The Ti-Al alloy structure with significant element proportions is shown in Figure 12. The experimental results are shown in Figure 13. The analysis of Figure 13 shows that when the content of Al remains unchanged, the increase in the content of V causes a slight change in the diffusion activation energy and a slight increase in the thickness of the oxide layer. However, when element V’s content is low, and element Al’s content increases, the diffusion activation energy of the oxygen element gradually increases, and the thickness of the oxide layer also changes significantly. It can be seen that the increase in the Al content has a more significant effect on the diffusion activation energy of oxygen than the increase in the V content, which, in turn, has a more significant effect on the thickness of the oxide layer.

## 4. Conclusions

In this study, the effect of cutting speed on the surface layer characteristics of titanium alloy Ti6Al4V was discussed, and the formation mechanism of the oxide layer and its effect on the surface peeling phenomenon was analyzed. The main conclusions of the study are summarized as follows:

1. With the increased cutting speed, the surface roughness increases first and then decreases under the detection scale more significantly than the feed amount (1.2 mm). It continues to increase under the detection scale, which is smaller than the feed amount (0.1 mm). This phenomenon reveals that the effect of cutting speed on the surface quality varies at different scales. With the increase in the cutting speed, the surface shedding area gradually increases, and the distribution of the red area (low roughness) changes from dispersion to concentration, reflecting the gradual deterioration of surface quality and the intensification of the shedding phenomenon.
2. The metamorphic layer’s thickness gradually increases as the cutting speed increases. When the cutting speed is 60 m/min, the surface layer of the newly formed material is not observed. When the velocity increases to 150 m/min, the thickness of the metamorphic layer is about 0.31 μm. When the velocity is further increased to 450 m/min, the thickness of the metamorphic layer increases to 0.714 μm. The increase in cutting speed leads to an increase in the cutting temperature, accelerates the oxidation reaction, and increases the thickness of the oxide layer. The binding force between the oxide layer and the matrix is weakened, and it is easy for this to fall off under the action of the cutting force, which leads to defects on the machined surface and affects the machining accuracy.
3. In this study, the first-principles calculation method established a prediction model of oxide layer thickness based on the diffusion activation energy of oxygen atoms in the Ti6Al4V alloy system. Compared with experimental data, it is verified that the prediction error of this model is less than 15%. Further calculation and analysis revealed that in the study investigating the influence of different element proportions on the thickness of the oxide layer, the increase of aluminum content had more significant effects on the diffusion activation energy of oxygen atoms and the thickness of the oxide layer than that of the vanadium content.

## Figures and Tables

**Table 1:** The experimental scheme.

Test Number	*v*_c_ (m/min)	*f* (mm/r)	*a*_p_ (mm)
1	60	0.2	0.2
2	150	0.2	0.2
3	250	0.2	0.2
4	350	0.2	0.2
5	450	0.2	0.2

**Table 2:** Material chemical composition of Ti6Al4V.

Ti (wt.%)	Al (wt.%)	V (wt.%)	Fe (wt.%)	C (wt.%)	N (wt.%)	H (wt.%)	O (wt.%)
Allowance	5.5~6.8	3.5~4.5	0.3	0.08	0.05	0.015	0.20

**Table 3:** Material performance parameters of Ti6Al4V (room temperature).

Yield Strength (*τ*)	Thermal Conductivity (λ)	Specific Heat (ϲ)	Density (ρ)	Thermal Softening Coefficient (α)
MPa	W(m·K)^−1^	J(kg·K)^−1^	kg/m^3^	1/K
825	6.8	520	4.44 × 10^3^	6.13 × 10^−4^

## Data Availability

The original contributions presented in the study are included in the article, further inquiries can be directed to the corresponding authors.
